# 4-(4-Meth­oxy­phen­yl)-2-methyl­but-3-yn-2-ol

**DOI:** 10.1107/S1600536810024529

**Published:** 2010-06-30

**Authors:** Frank Eissmann, Uwe Kafurke, Edwin Weber

**Affiliations:** a Institut für Organische Chemie, TU Bergakademie Freiberg, Leipziger Str. 29, D-09596 Freiberg/Sachsen, Germany

## Abstract

The mol­ecular structure of the title compound, C_12_H_14_O_2_, features a nearly coplanar arrangement including the aromatic ring, the C C—C group and the ether O atom. The maximum deviation from the least-squares plane of these ten atoms is 0.0787 (8) Å for the ether O atom. In the crystal, mol­ecules are connected *via* O—H⋯O hydrogen bonds (involving the hy­droxy O atom both as hydrogen-bond donor and acceptor) and weaker (ar­yl)C—H⋯π(ar­yl) contacts, leading to the formation of strands running parallel to the *b* axis. Further stabilization results from weaker (meth­yl)C—H⋯π(acetyl­ene) inter­actions between different strands.

## Related literature

For general background to the Sonogashira–Hagihara coupling reaction and for applications of terminal aryl­alkynes, see: Chinchilla & Nájera (2007 ▶); Sonogashira (1998 ▶). For an alternative synthesis of the title compound, also including analytical data, see: Mayr & Halberstadt-Kausch (1982 ▶). For C–H⋯π hydrogen bonding, see: Nishio *et al.* (2009 ▶).
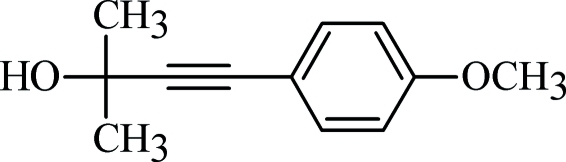


## Experimental

### 

#### Crystal data

C_12_H_14_O_2_
*M* *_r_* = 190.23
Orthorhombic, 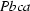

*a* = 16.0390 (13) Å
*b* = 5.8399 (5) Å
*c* = 22.5298 (19) Å
*V* = 2110.3 (3) Å^3^
*Z* = 8
Mo *K*α radiation
μ = 0.08 mm^−1^
*T* = 153 K
0.60 × 0.28 × 0.24 mm

#### Data collection

Bruker Kappa APEXII CCD diffractometer
Absorption correction: multi-scan (*SADABS*; Bruker, 2007 ▶) *T*_min_ = 0.928, *T*_max_ = 0.981
39990 measured reflections
1956 independent reflections
1662 reflections with *I* > 2σ(*I*)
*R*_int_ = 0.025

#### Refinement

*R*[*F* ^2^ > 2σ(*F* ^2^)] = 0.037
*wR*(*F* ^2^) = 0.114
*S* = 1.10
1956 reflections
131 parameters
H-atom parameters constrained
Δρ_max_ = 0.23 e Å^−3^
Δρ_min_ = −0.21 e Å^−3^

### 

Data collection: *APEX2* (Bruker, 2007 ▶); cell refinement: *SAINT* (Bruker, 2007 ▶); data reduction: *SAINT*; program(s) used to solve structure: *SHELXS97* (Sheldrick, 2008 ▶); program(s) used to refine structure: *SHELXL97* (Sheldrick, 2008 ▶); molecular graphics: *SHELXTL* (Sheldrick, 2008 ▶) and *ORTEP-3* (Farrugia, 1997 ▶); software used to prepare material for publication: *SHELXTL* and *PLATON* (Spek, 2009 ▶).

## Supplementary Material

Crystal structure: contains datablocks I, global. DOI: 10.1107/S1600536810024529/sj5028sup1.cif

Structure factors: contains datablocks I. DOI: 10.1107/S1600536810024529/sj5028Isup2.hkl

Additional supplementary materials:  crystallographic information; 3D view; checkCIF report

## Figures and Tables

**Table 1:** Hydrogen-bond geometry (Å, °) *Cg*1 and π2 are the centroid of the C1–C6 aromatic ring and the midpoint of the C8 C9 bond, respectively.

*D*—H⋯*A*	*D*—H	H⋯*A*	*D*⋯*A*	*D*—H⋯*A*
O2—H1⋯O2^i^	0.84	2.31	3.1463 (7)	178
C5—H5⋯*Cg*1^i^	0.95	2.96	3.7452 (12)	141
C11—H11*B*⋯*π*2^ii^	0.98	2.80	3.7443 (14)	161

Symmetry codes: (i) 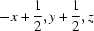; (ii) 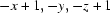.

**Fig. 1.**
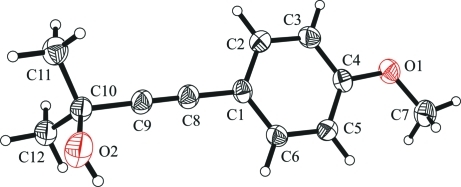
The molecular structure of the title compound, showing the atom numbering scheme. Displacement ellipsoids are drawn at the 50% probability level.

**Fig. 2.**
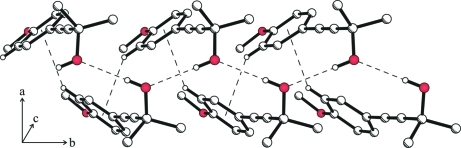
Molecular strands within the packing of the title compound. Hydrogen bonds are represented as dashed lines. H atoms not involved in any hydrogen bond have been omitted for clarity.

**Fig. 3.**
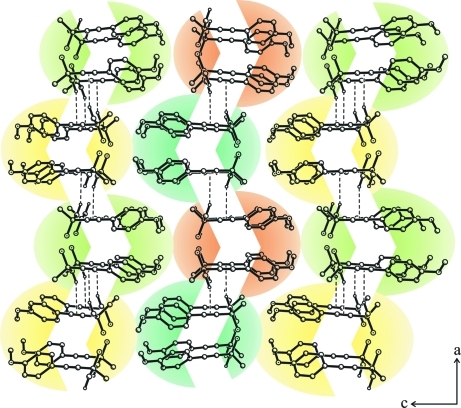
Packing diagram viewed down the b axis, showing the antiparallel orientation of the strands (symbolized by different background shading) as well as the resulting zigzag layers. The C11–H11B···Cg02 interactions between different strands are represented as dashed lines. H atoms not involved in hydrogen bonding between the strands have been omitted.

## supplementary crystallographic information

### Comment

Terminal arylalkynes are of general interest in organic chemistry as they can be used for subsequent coupling reactions leading to diarylalkynes which are important building blocks in materials science (Chinchilla & Nájera, 2007). Compounds of this type can be prepared in a two-step reaction where an aryl halide is reacted with 2-methylbut-3-yn-2-ol (a monoprotected acetylene) in a Sonogashira-Hagihara reaction followed by deprotection of the resulting acetylenic compound using a base-catalysed retro-Favorsky elimination of acetone (Sonogashira, 1998).

Following this strategy, the title compound, which is important as an intermediate for the preparation of the terminal arylalkyne 4-ethinylanisole, was prepared *via* a Sonogashira-Hagihara coupling reaction starting from 4-bromoanisole and 2-methylbut-3-yn-2-ol. Crystallization from cyclohexane yielded colourless needles suitable for an X-ray crystal structure analysis on which is reported herein.

The title compound, C_12_H_14_O_2_, crystallizes in the orthorhombic space group *Pbca*. The asymmetric unit consists of one molecule which is illustrated in Fig. 1. The atoms C1–C6, C8–C10 and O1 are arranged nearly coplanar. Only the ether O atom O1 deviates slightly from the least-squares plane involving the mentioned atoms. The deviation between this least-squares plane and O1 is 0.0787 (8) Å, the deviation of all other atoms ranges from 0.0023 (11) to 0.0390 (10) Å. The methoxy methyl group is only marginally distorted towards this least-squares plane (torsion angles of -2.84 (18)° and 176.38 (11)° for C5–C4–O1–C7 and C3–C4–O1–C7, respectively). The angle between the least-squares plane (C1–C6, C8–C10, O1) and the least-squares plane of the atoms C9, C10 and O2 is 40.60 (11)°.

In the crystal, the hydroxy O atom O2 is involved in an O2–H1···O2 hydrogen bond leading to the formation of one-dimensional strands located parallel to the *b* axis (Fig. 2). Within these strands further stabilization is reached *via* a weak C–H···π interaction (Nishio *et al.*, 2009) of the type (aryl)C–H···π(aryl) between C5–H5 and *Cg*1 (*Cg*1 corresponds to the centroid of the aromatic ring). Within the packing, these one-dimensional strands are organized antiparallel, which is shown in Fig. 3. Different strands are connected *via* a weak (methyl)C–H···π(acetylene) contact involving C11–H11B and π2 (π2 is the midpoint of the C≡C bond). This interaction leads to a connection of different strands resulting in the formation of zigzag layers parallel to the *ab* plane (Fig. 3).

### Experimental

The title compound was prepared as follows: To a degassed mixture of 22.44 g (0.12 mol) 4-bromoanisole and 12.20 g (0.145 mol) 2-methylbut-3-yn-2-ol in 100 ml of diethylamine were added 0.27 g (1.2 mmol) palladium(II) acetate, 0.63 g (2.4 mmol) triphenylphosphane and 0.11 g (0.6 mmol) copper(I) iodide. The resulting mixture was refluxed for 15 h under argon, followed by a second addition of the catalyst mixture (same amounts as before) and another 15 h refluxing under argon. The solvent was removed *in vacuo* and the residue dissolved in water. The aqueous solution was extracted several times with diethyl ether, the combined organic phases dried over Na_2_SO_4_ and concentrated *in vacuo*. Column chromatographic purification [1. Al_2_O_3_, activity 1, eluent: diethyl ether; 2. silica gel, eluent: *n*-pentane/ethyl acetate (6:1 *v/v*)] and crystallization from cyclohexane yielded the title compound as colourless needles (16.8 g, 74% yield, m.p. 326 K). The analytical data are in agreement with the literature (Mayr & Halberstadt-Kausch, 1982), where the synthesis of the title compound is described using another procedure.

### Refinement

H atoms were positioned geometrically and allowed to ride on their respective parent atoms, with C—H = 0.98 Å and *U*_iso_(H) = 1.5 *U*_eq_(C) for methyl, C—H = 0.95 Å and *U*_iso_(H) = 1.2 *U*_eq_(C) for aryl and O—H = 0.84 Å and *U*_iso_(H) = 1.5 *U*_eq_(O) for hydroxy H atoms.

### Crystal data

**Table:**

C_12_H_14_O_2_	*D*_x_ = 1.198 Mg m^−^^3^
*M**_r_* = 190.23	Melting point: 326 K
Orthorhombic, *P**b**c**a*	Mo *K*α radiation, λ = 0.71073 Å
Hall symbol: -P 2ac 2ab	Cell parameters from 9596 reflections
*a* = 16.0390 (13) Å	θ = 2.5–35.7°
*b* = 5.8399 (5) Å	µ = 0.08 mm^−^^1^
*c* = 22.5298 (19) Å	*T* = 153 K
*V* = 2110.3 (3) Å^3^	Piece, colourless
*Z* = 8	0.60 × 0.28 × 0.24 mm
*F*(000) = 816	

### Data collection

**Table:**

Bruker Kappa APEXII CCD diffractometer	1956 independent reflections
Radiation source: fine-focus sealed tube	1662 reflections with *I* > 2σ(*I*)
graphite	*R*_int_ = 0.025
φ and ω scans	θ_max_ = 25.5°, θ_min_ = 2.2°
Absorption correction: multi-scan (*SADABS*; Bruker, 2007)	*h* = −19→19
*T*_min_ = 0.928, *T*_max_ = 0.981	*k* = −7→7
39990 measured reflections	*l* = −27→27

### Refinement

**Table:**

Refinement on *F*^2^	Primary atom site location: structure-invariant direct methods
Least-squares matrix: full	Secondary atom site location: difference Fourier map
*R*[*F*^2^ > 2σ(*F*^2^)] = 0.037	Hydrogen site location: inferred from neighbouring sites
*wR*(*F*^2^) = 0.114	H-atom parameters constrained
*S* = 1.10	*w* = 1/[σ^2^(*F*_o_^2^) + (0.0611*P*)^2^ + 0.5536*P*] where *P* = (*F*_o_^2^ + 2*F*_c_^2^)/3
1956 reflections	(Δ/σ)_max_ < 0.001
131 parameters	Δρ_max_ = 0.23 e Å^−^^3^
0 restraints	Δρ_min_ = −0.21 e Å^−^^3^

### Special details

**Table:**

Geometry. All e.s.d.'s (except the e.s.d. in the dihedral angle between two l.s. planes) are estimated using the full covariance matrix. The cell e.s.d.'s are taken into account individually in the estimation of e.s.d.'s in distances, angles and torsion angles; correlations between e.s.d.'s in cell parameters are only used when they are defined by crystal symmetry. An approximate (isotropic) treatment of cell e.s.d.'s is used for estimating e.s.d.'s involving l.s. planes.
Refinement. Refinement of *F*^2^ against ALL reflections. The weighted *R*-factor *wR* and goodness of fit *S* are based on *F*^2^, conventional *R*-factors *R* are based on *F*, with *F* set to zero for negative *F*^2^. The threshold expression of *F*^2^ > σ(*F*^2^) is used only for calculating *R*-factors(gt) *etc*. and is not relevant to the choice of reflections for refinement. *R*-factors based on *F*^2^ are statistically about twice as large as those based on *F*, and *R*- factors based on ALL data will be even larger.

### Fractional atomic coordinates and isotropic or equivalent isotropic displacement parameters (Å^2^)

**Table:**

	*x*	*y*	*z*	*U*_iso_*/*U*_eq_	
O1	0.37704 (6)	0.71897 (17)	0.22519 (4)	0.0376 (3)	
O2	0.28653 (6)	0.0689 (2)	0.57437 (5)	0.0557 (4)	
H1	0.2669	0.2022	0.5734	0.084*	
C1	0.37656 (7)	0.4050 (2)	0.39090 (5)	0.0278 (3)	
C2	0.42516 (8)	0.3244 (2)	0.34371 (5)	0.0336 (3)	
H2	0.4587	0.1918	0.3490	0.040*	
C3	0.42501 (8)	0.4349 (2)	0.28967 (5)	0.0343 (3)	
H3	0.4587	0.3790	0.2582	0.041*	
C4	0.37571 (7)	0.6277 (2)	0.28114 (5)	0.0282 (3)	
C5	0.32853 (8)	0.7139 (2)	0.32768 (5)	0.0311 (3)	
H5	0.2958	0.8480	0.3224	0.037*	
C6	0.32961 (7)	0.6019 (2)	0.38213 (5)	0.0310 (3)	
H6	0.2975	0.6614	0.4140	0.037*	
C7	0.32340 (10)	0.9090 (3)	0.21383 (6)	0.0427 (4)	
H7A	0.2656	0.8654	0.2224	0.064*	
H7B	0.3283	0.9541	0.1721	0.064*	
H7C	0.3395	1.0378	0.2393	0.064*	
C8	0.37456 (7)	0.2863 (2)	0.44679 (5)	0.0310 (3)	
C9	0.37190 (7)	0.1876 (2)	0.49347 (5)	0.0317 (3)	
C10	0.37033 (7)	0.0710 (2)	0.55174 (5)	0.0313 (3)	
C11	0.39535 (9)	−0.1775 (2)	0.54539 (6)	0.0374 (3)	
H11A	0.3914	−0.2535	0.5841	0.056*	
H11B	0.4528	−0.1867	0.5309	0.056*	
H11C	0.3580	−0.2534	0.5171	0.056*	
C12	0.42831 (10)	0.1924 (2)	0.59512 (6)	0.0410 (3)	
H12A	0.4124	0.3540	0.5981	0.061*	
H12B	0.4859	0.1809	0.5809	0.061*	
H12C	0.4239	0.1202	0.6343	0.061*	

### Atomic displacement parameters (Å^2^)

**Table:**

	*U*^11^	*U*^22^	*U*^33^	*U*^12^	*U*^13^	*U*^23^
O1	0.0414 (5)	0.0451 (6)	0.0264 (5)	0.0022 (4)	0.0057 (4)	0.0083 (4)
O2	0.0300 (5)	0.0844 (9)	0.0528 (6)	0.0158 (5)	0.0129 (4)	0.0270 (6)
C1	0.0240 (6)	0.0340 (6)	0.0255 (6)	−0.0028 (4)	−0.0030 (4)	0.0019 (5)
C2	0.0319 (6)	0.0356 (7)	0.0332 (7)	0.0071 (5)	−0.0018 (5)	0.0000 (5)
C3	0.0312 (6)	0.0435 (7)	0.0281 (6)	0.0046 (5)	0.0047 (5)	−0.0021 (5)
C4	0.0271 (6)	0.0332 (6)	0.0245 (6)	−0.0059 (5)	0.0000 (4)	0.0022 (5)
C5	0.0330 (6)	0.0309 (6)	0.0294 (6)	0.0041 (5)	0.0009 (5)	0.0016 (5)
C6	0.0293 (6)	0.0378 (7)	0.0257 (6)	0.0030 (5)	0.0028 (4)	−0.0003 (5)
C7	0.0548 (9)	0.0405 (8)	0.0328 (7)	0.0018 (6)	0.0000 (6)	0.0120 (6)
C8	0.0260 (6)	0.0366 (7)	0.0304 (6)	0.0016 (5)	−0.0022 (4)	0.0019 (5)
C9	0.0261 (6)	0.0381 (7)	0.0308 (7)	0.0049 (5)	−0.0013 (4)	0.0039 (5)
C10	0.0240 (6)	0.0399 (7)	0.0298 (6)	0.0050 (5)	0.0037 (4)	0.0078 (5)
C11	0.0412 (7)	0.0330 (7)	0.0379 (7)	−0.0044 (5)	0.0004 (5)	0.0048 (5)
C12	0.0576 (9)	0.0336 (7)	0.0317 (7)	0.0058 (6)	−0.0048 (6)	0.0012 (5)

### Geometric parameters (Å, °)

**Table:**

O1—C4	1.3687 (14)	C6—H6	0.9500
O1—C7	1.4271 (17)	C7—H7A	0.9800
O2—C10	1.4376 (14)	C7—H7B	0.9800
O2—H1	0.8400	C7—H7C	0.9800
C1—C6	1.3885 (18)	C8—C9	1.2000 (18)
C1—C2	1.3998 (17)	C9—C10	1.4791 (16)
C1—C8	1.4379 (17)	C10—C11	1.5125 (18)
C2—C3	1.3778 (17)	C10—C12	1.5239 (19)
C2—H2	0.9500	C11—H11A	0.9800
C3—C4	1.3895 (18)	C11—H11B	0.9800
C3—H3	0.9500	C11—H11C	0.9800
C4—C5	1.3875 (17)	C12—H12A	0.9800
C5—C6	1.3902 (17)	C12—H12B	0.9800
C5—H5	0.9500	C12—H12C	0.9800
			
C4—O1—C7	117.28 (10)	O1—C7—H7C	109.5
C10—O2—H1	109.5	H7A—C7—H7C	109.5
C6—C1—C2	118.19 (11)	H7B—C7—H7C	109.5
C6—C1—C8	120.78 (10)	C9—C8—C1	179.24 (12)
C2—C1—C8	121.03 (11)	C8—C9—C10	178.32 (13)
C3—C2—C1	120.84 (11)	O2—C10—C9	109.54 (10)
C3—C2—H2	119.6	O2—C10—C11	105.87 (11)
C1—C2—H2	119.6	C9—C10—C11	110.70 (10)
C2—C3—C4	120.18 (11)	O2—C10—C12	110.31 (11)
C2—C3—H3	119.9	C9—C10—C12	110.16 (10)
C4—C3—H3	119.9	C11—C10—C12	110.18 (10)
O1—C4—C5	124.29 (11)	C10—C11—H11A	109.5
O1—C4—C3	115.72 (10)	C10—C11—H11B	109.5
C5—C4—C3	119.99 (11)	H11A—C11—H11B	109.5
C4—C5—C6	119.30 (11)	C10—C11—H11C	109.5
C4—C5—H5	120.4	H11A—C11—H11C	109.5
C6—C5—H5	120.4	H11B—C11—H11C	109.5
C1—C6—C5	121.46 (11)	C10—C12—H12A	109.5
C1—C6—H6	119.3	C10—C12—H12B	109.5
C5—C6—H6	119.3	H12A—C12—H12B	109.5
O1—C7—H7A	109.5	C10—C12—H12C	109.5
O1—C7—H7B	109.5	H12A—C12—H12C	109.5
H7A—C7—H7B	109.5	H12B—C12—H12C	109.5
			
C6—C1—C2—C3	−1.33 (18)	C2—C3—C4—C5	2.07 (18)
C8—C1—C2—C3	178.08 (11)	O1—C4—C5—C6	177.57 (11)
C1—C2—C3—C4	−0.57 (19)	C3—C4—C5—C6	−1.62 (18)
C7—O1—C4—C5	−2.84 (18)	C2—C1—C6—C5	1.78 (17)
C7—O1—C4—C3	176.38 (11)	C8—C1—C6—C5	−177.63 (11)
C2—C3—C4—O1	−177.19 (11)	C4—C5—C6—C1	−0.33 (18)

### Hydrogen-bond geometry (Å, °)

**Table:**

*Cg*1 and π2 are the centroid of the C1–C6 aromatic ring and midpoint of the C8≡C9 bond, respectively.

**Table:**

*D*—H···*A*	*D*—H	H···*A*	*D*···*A*	*D*—H···*A*
O2—H1···O2^i^	0.84	2.31	3.1463 (7)	178
C5—H5···Cg1^i^	0.95	2.96	3.7452 (12)	141
C11—H11B···π2^ii^	0.98	2.80	3.7443 (14)	161

Symmetry codes: (i) −*x*+1/2, *y*+1/2, *z*; (ii) −*x*+1, −*y*, −*z*+1.
